# Real-time detection of particleboard surface defects based on improved YOLOV5 target detection

**DOI:** 10.1038/s41598-021-01084-x

**Published:** 2021-11-05

**Authors:** Ziyu Zhao, Xiaoxia Yang, Yucheng Zhou, Qinqian Sun, Zhedong Ge, Dongfang Liu

**Affiliations:** 1 School of Information and Electrical Engineering, Shandong Jianzhu University, Jinan, 250101 China; 2 Department of Quality Control, Shandong Institute for Quality Inspection, Jinan, 250000 China

**Keywords:** Engineering, Electrical and electronic engineering

## Abstract

Particleboard surface defect detection technology is of great significance to the automation of particleboard detection, but the current detection technology has disadvantages such as low accuracy and poor real-time performance. Therefore, this paper proposes an improved lightweight detection method of You Only Live Once v5 (YOLOv5), namely PB-YOLOv5 (Particle Board-YOLOv5). Firstly, the gamma-ray transform method and the image difference method are combined to deal with the uneven illumination of the acquired images, so that the uneven illumination is well corrected. Secondly, Ghost Bottleneck lightweight deep convolution module is added to Backbone module and Neck module of YOLOv5 detection algorithm to reduce model volume. Thirdly, the SELayer module of attention mechanism is added into Backbone module. Finally, replace Conv in Neck module with depthwise convolution (DWConv) to compress network parameters. The experimental results show that the PB-YOLOv5 model proposed in this paper can accurately identify five types of defects on the particleboard surface: Bigshavings, SandLeakage, GlueSpot, Soft and OliPollution, and meet the real-time requirements. Specifically, recall, F1 score, mAP@.5, mAP@.5:.95 values of pB-Yolov5s model were 91.22%, 94.5%, 92.1%, 92.8% and 67.8%, respectively. The results of Soft defects were 92.8%, 97.9%, 95.3%, 99.0% and 81.7%, respectively. The detection of single image time of the model is only 0.031 s, and the weight size of the model is only 5.4 MB. Compared with the original YOLOv5s, YOLOv4, YOLOv3 and Faster RCNN, the PB-Yolov5s model has the fastest Detection of single image time. The Detection of single image time was accelerated by 34.0%, 55.1%, 64.4% and 87.9%, and the weight size of the model is compressed by 62.5%, 97.7%, 97.8% and 98.9%, respectively. The mAP value increased by 2.3%, 4.69%, 7.98% and 13.05%, respectively. The results show that the PB-YOLOV5 model proposed in this paper can realize the rapid and accurate detection of particleboard surface defects, and fully meet the requirements of lightweight embedded model.

## Introduction

Particleboard is a wood-based board made by gluing wood or other lignocellulosic materials into scrap material. Due to the influence of many factors such as raw material quality and production technology in the production process of continuous chipboard press, there will be some defects on the surface of some products, including OliPollution, GlueSpot, BigShavings, Soft, SandLeakage and other common types^1,2^. Surface defects will reduce the strength of the plate, affect the appearance of the plate, and bring difficulties to the secondary processing^3^. At present, the detection of surface defects of particleboard is still in the stage of artificial eye detection, and the duration of artificial visual inspection varies from person to person. On the one hand, it is difficult to accurately measure the area and size of defects by visual inspection to millimeter level. Long-term visual observation can not ensure the accuracy of detection, and missed detection and wrong judgment often occur. On the other hand, according to the communication between the staff of HuiFeng wood company and FengLin company, the speed of manual visual inspection was lower than that of the production line, so the production line can only cooperate with manual visual inspection in low-speed operation mode, which seriously reduces the delivery efficiency of particleboard.

Therefore, the mechanization and intellectualization of particleboard surface defect detection was a major problem of particleboard defect detection all over the world^4^.

Because the surface defect detection of plate has a very strong application, experts and scholars of various countries have carried out a variety of research on the surface defect detection. Most of the research objects are carried out on the surface defects of wood and veneer, while the surface defects of particleboard are rarely studied. For example, Chacon et al. used the fuzzy self-organizing neural network as a classifier to detect the surface defects of wood veneer, and the identification accuracy was 91.17%. Although the accuracy meets the requirements, the detection time still cannot meet the requirements of the production line^5^. He et al. proposed a hybrid full convolutional neural network to detect the location of wood surface defects and classify defect types from wood surface images, with an identification rate of 91.31%. The method was still being studied in the laboratory and was still a long way from the requirements of the production line^6^. Xie et al. proposed a defect detection method based on the surface texture features of mixed wood, with an average detection time of 1.83s and an accuracy rate of 92.67%. In the actual detection of surface defects, it will be affected by noise and other external influences, and the identification rate of GlueSpot and OliPollution was low^7^. Zhang et al. took wood defects as the research object and used BP neural network to identify the defects, and the recognition rate reached 93%^8^. Li et al. proposed a method based on local binary pattern (LBP) for classification and detection of birch veneer cracks, and the classification accuracy reached 94%. The algorithm of LBP was relatively simple, and its generalization ability was low, so it can only be applied to the detection of wood veneer. Moreover, the detection speed of LBP method was relatively slow and cannot adapt to the detection speed of the production line^9^. Guo et al. proposed an algorithm for surface defect extraction of particleboard based on gray level co-occurrence matrix hierarchical clustering. According to the different texture characteristics of defect area and normal area, the hierarchical clustering algorithm was used to extract defects, and the accuracy of defect extraction reached 92.2%. The running time of this algorithm is long and can not meet the real time demand^10^.

At present, most studies focus on improving the accuracy of image segmentation and defect classification, while there were few studies on improving detection speed and real-time detection algorithm, and most studies do not have detailed data on algorithm execution time^11–15^. To solve the existing problems, this paper proposes a lightweight YOLOv5 model named PB-YOLOv5. Compared with the standard YOLOv5 model, the number of parameters and operation cost of this model were reduced, the weight of the model was reduced, and the operation speed was greatly reduced. Compared with the previous results, the main contributions of this paper can be summarized as follows:

1. A new PB-YOLOv5 method for particleboard surface defect detection was proposed. In order to reduce the computing cost and meet the design of lightweight neural network, the model is easier to deploy mobile devices and embedded devices.
2. Introduce Ghost Bottleneck module and SELayer model into Backbone model and Neck module in YOLOv5s. And replace the ordinary Convolution layer of the original PANet module with Depthwise Convolution of the Neck module.
3. A correction method based on the combination of gamma transform and image difference is proposed to solve the illumination unevenness of the original image in the plate region.
4. Experiments on particleboard surface defect detection data set show that PB-YOLOv5 has better index results than the existing target detection methods.

## Materials and methods

### Test equipment

**Figure 1:**
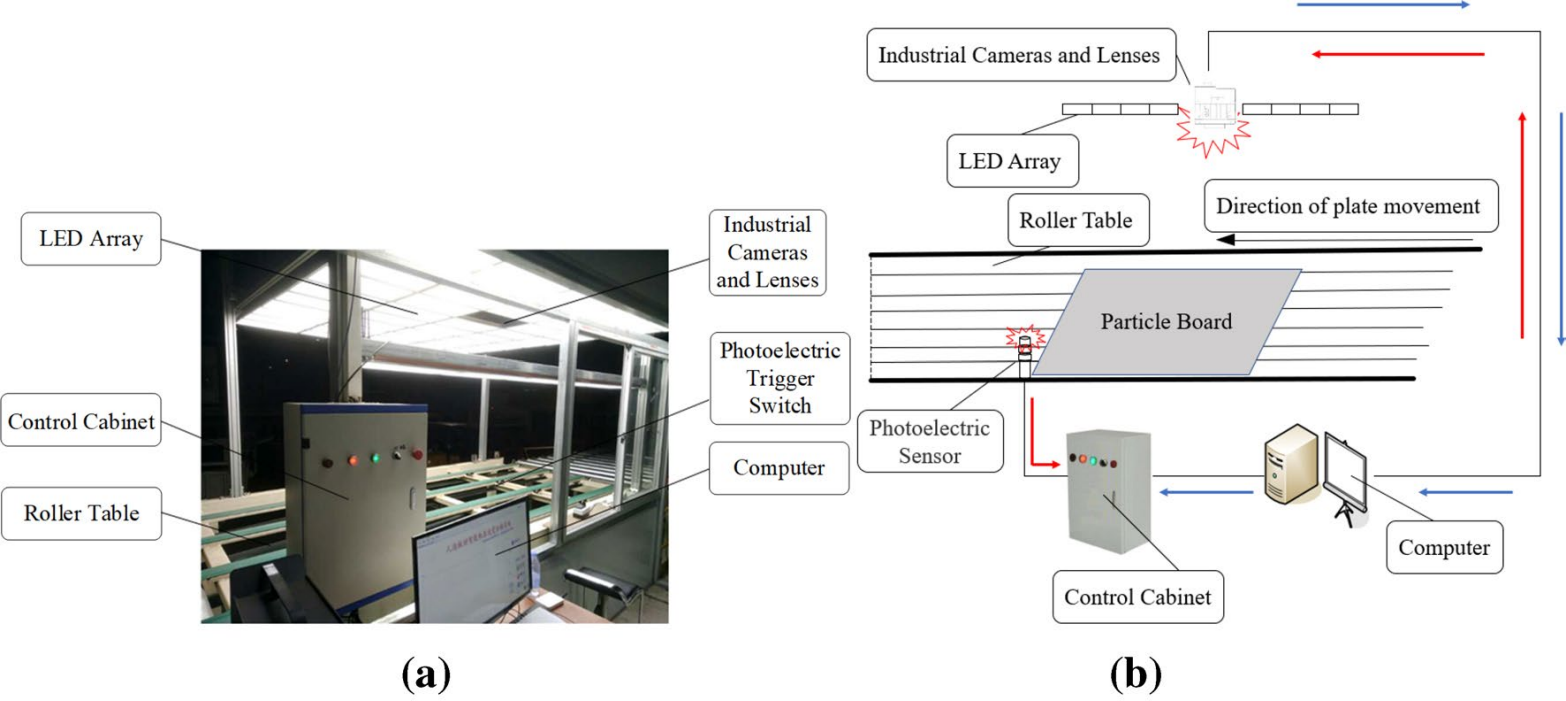
Data acquisition equipment to collect original images of surface defects on particleboard: (**a**) system hardware composition, (**b**) schematic diagram of image acquisition process.

The team independently developed a set of particleboard surface defect detection equipment. The hardware composition of the defect detection system is shown in Fig. 1a. The camera selection was SP-5000 M-CXP4-USB 3.0 high performance industrial CMOS array camera produced by JAI Company of Denmark. The relevant parameters of the camera are shown in Table 1.

**Table 1:** Camera and hardware related parameters.

Parameter name	Parameter value	Parameter name	Parameter value
Sensor	CMOS	Exposure time	10 µs ~ 8 s
Resolution (H × V)	2560pix × 2048pix	Visual output	8/10/12 bit
Frames	62 fps	Working conditions	− 45 °C ~ + 70 °C

The schematic diagram of the image acquisition process is shown in Fig. 1b. The red line path was when the particleboard was conveyed to the photoelectric sensor by the production line, the photoelectric sensor sends a signal to the PLC controller and triggers the camera to take pictures. The blue line path was to transmit the whole image of particleboard to the computer to complete the acquisition of one image. The acquisition speed is 0.3003s/piece, and the size of each image is 2560pix × 2048pix. The program automatically crops the obtained particleboard image into 1751pix × 911pix, keeping only the area of interest and cutting off the redundant parts.

### The data collection

For particleboard surface defect detection requirements of high precision and high real-time. Specifically, five types of defects should be identified on the image to be tested. The five types of defects are BigShavings, SandLeakage, GlueSpot, Soft and OliPollution. Among them, BigShavings, OliPollution, GlueSpot and Soft are characterized by small area, but the difference between the gray value of defect and that of normal plate surface is more than 50, which is easy to distinguish. SandLeakage defect was characterized by large area, but the difference between gray value and normal plate gray value is very small, which is often confused with the normal area with uneven sanding. In the study, 879 images with surface defects were manually screened from 420,000 particleboard images.

### Solve the phenomenon of uneven illumination

**Figure 2:**
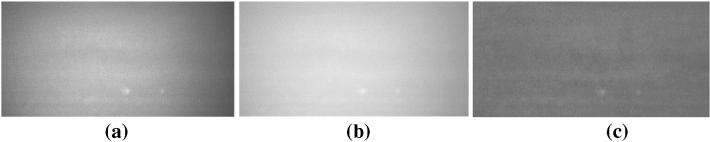
Comparison of illumination nonuniformity before and after correction: (**a**) original image of particleboard, (**b**) gamma transformation result, (**c**) gamma transform and image difference result images.


Because the site of this study was affected by camera performance, light source and natural lighting environment, etc. It will cause uneven illumination in the extracted panel area. Therefore, a method of gamma transform and image difference was adopted to solve the illumination inhomogeneity in the process of data collection and improve the accuracy of subsequent detection. Algorithm.1 was the process of solving illumination inhomogeneity, and the comparison of illumination inhomogeneity of images before and after correction was shown in Fig. 2. Figure 2b was the result of only used gamma transform. The uneven illumination is not completely eliminated, and the edge region of the plate is still slightly darker than the middle region. As shown in Fig. 2c, the brightness of the whole image is consistent, the uneven illumination of the original image is well corrected, and the shape of the defect is well maintained.

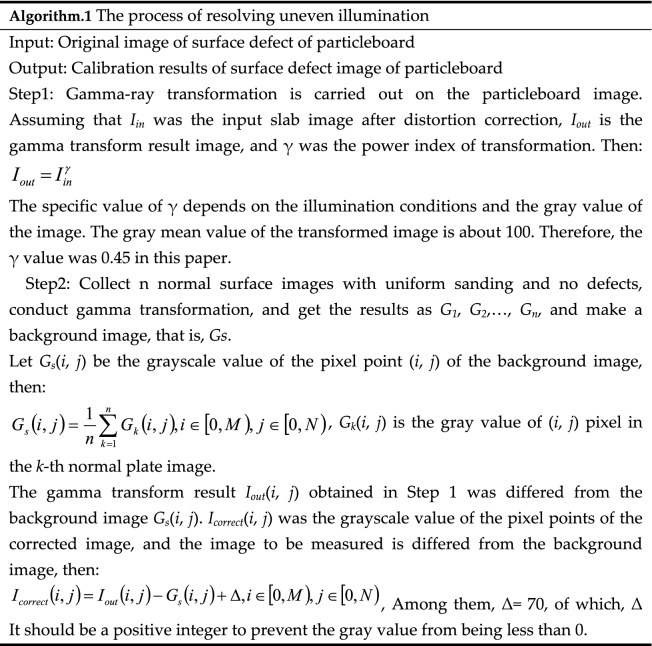


### Dataset preprocessing

Since the shapes of similar defects on the particleboard surface were different, whether the neural network can correctly process the collected defect images depends on the integrity of the training dataset. Therefore, the col-lected images were preprocessed by gray transformation, adding noise, rotation, median filtering, linear spatial filtering, etc., and the dataset was expanded from 879 to 6776 images. The enhanced images can be put into neural network for training, which can improve the generalization ability of the model, reduce the occurrence of overfitting phenomenon, and reduce the influence of additional factors on recognition.

Firstly, 80% of the dataset is used as the training data of PB-YOLOv5 target detection algorithm. Then, the remaining 20% is used as test data. LabelImg was used to manually mark the sur-face defects in the 6776 images, and ensure that each surface defect was accurately marked during the marking, with a total of 9676 defects were marked, as shown in Table 2. The probability of multiple defects appearing on the same particleboard was high.

**Table 2:** Dataset information of particleboard surface defects.

Dataset number	Training and testing dataset number	Defect name	Defect number
6776 images	Training dataset (5421 images)	BigShavings	1459 defects
		Soft	2708 defects
		OilPollution	1005 defects
		GlueSpot	1941 defects
		SandLeakage	627 defects
	Testing dataset (1355 images)	BigShavings	365 defects
		Soft	678 defects
		OilPollution	251 defects
		GlueSpot	485 defects
		SandLeakage	157 defects

### Original YOLOv5s network architecture

General architecture of object detection network adopted by YOLOv5 model. The network structure was divided into four parts according to the processing stage, including input, Backbone, Neck and prediction. The Backbone module was composed of Focus structure, Bottleneck layer, BottleneckCSP and SPP structure (space pyramid pooling).

The Backbone module was mainly composed of Bottleneck to reduce and expand the number of channels. The specific approach was to first reduce the channel by half through 1 × 1 convolution, and then double the number of channels through 3 × 3 convolution to obtain features, with the number of channels of input and output unchanged. The Neck module mainly adopts the PANet structure (Path Aggregation Network). The feature extractor of the module adopted a new enhanced bottom-up path, namely FPN structure (Feature Pyramid Networks), with improved the propagation of low-level features.

### Improvment of YOLOv5s network architecture design (PB-YOLO5s)

According to different network depth and width, YOLOv5 can be divided into four basic network structures: YOLOv5s, YOLOv5m, YOLOv5l and YOLOv5x^16,17^. The model size and the number of model parameters of the four architectures increase successively. Considering that particle board defect detection algorithm needs to meet the real-time requirements of production line, this study focuses on improved the designed of YOLOv5s architecture.

The principle of this model is to solve the object detection as a regression problem, which can predict the position and category of multiple target boxes at the same time. The end-to-end method is used for target detection and recognition, so as to realize the regression function. Without complex design process, the whole feature map can be directly selected for model training, which can better distinguish the target and background area. It has the characteristics of high positioning accuracy and fast detection speed. Particleboard surface defect detection and recognition algorithm needs to consider the deployment of the model on mobile devices. At present, the computational power of mobile devices and embedded devices is too low, and the existing deep convolution model is not practical to be directly used on embedded devices. Therefore, it is necessary to design a lightweight deep neural network model to solve the current problems.

**Figure 3:**
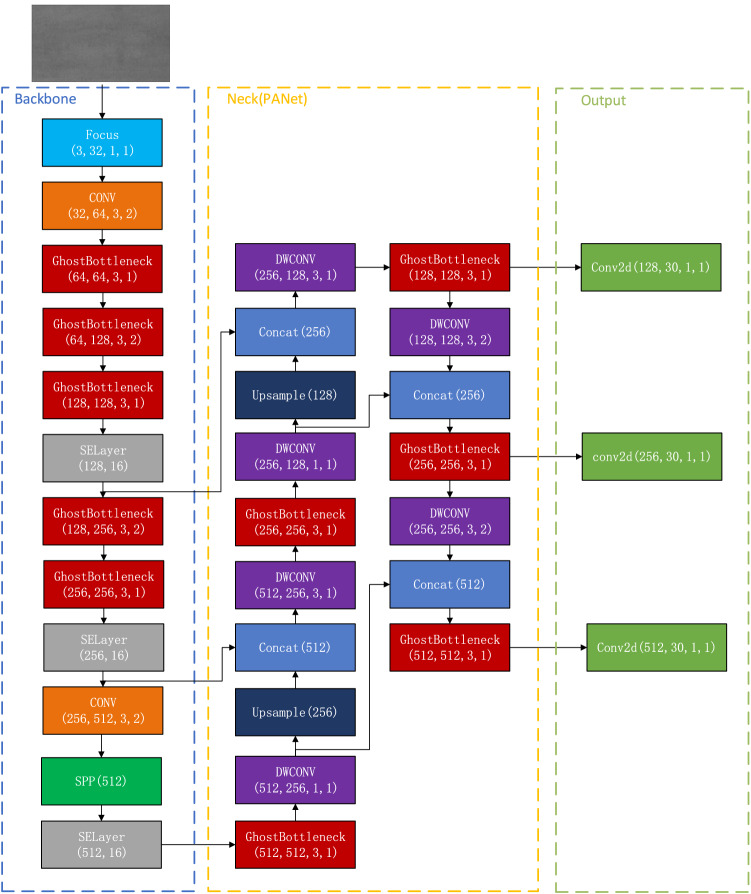
Modified Yolov5s structure (PB-Yolov5s).

In this study, GhostBottleneck module is introduced into the Backbone model and neck module in YOLOv5s, and SELayer is added. The purpose is to compress the network parameters as much as possible, which can further reduce the amount of network calculation and greatly improve the model reasoning speed. And replace the Conv layer of the original PANet module with DWConv layer in the Neck module. Compared with conventional convolution operation, the number of parameters and operation cost were greatly reduced, and spatial feature information of different sizes can be extracted, which improves the robustness of the model for spatial layout and object recognition. Figure 3 shows the schematic diagram of the improved YOLOv5s structure, where the parameters in brackets of each box are respectively the output size of the feature map of the previous layer as the input size of the feature map of this layer, the output size of the feature map of this layer, the size of the convolution kernel and the size of the step size.

### Improvement of backbone network

**Figure 4:**
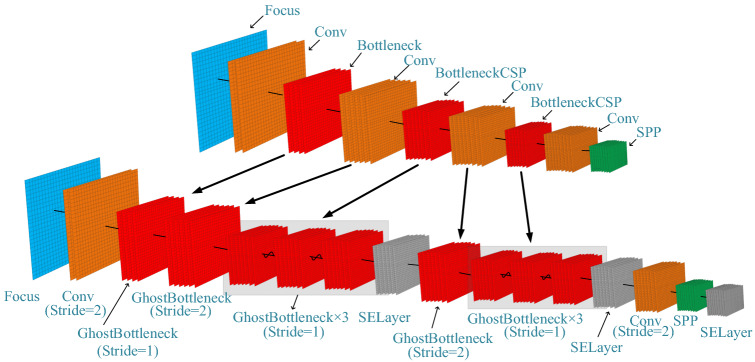
Improved BackBone module.

BottleneckCSP and CONV in the Backbone module were replaced with Ghost Bottleneck module, as shown in Fig. 4. Replace the Bottleneck of the third layer with the Stride = 1 of Ghost Bottleneck, and the output size of the model remains the same. Replace the Conv of layer 4 and layer 6 with the Stride = 2 Ghost Bottleneck for the following sampling processing. It has the purpose of dimensionality reduction, reducing the number of parameters to be learned in the network, and expanding the receptive field to prevent the occurrence of overfitting. BottleneckCSP modules at the fifth and seventh levels were changed into three Ghost Bottleneck modules with a Stride = 1. The SELayer module is added after the feature map of each scale, so that the depth of the feature map of the previous layer is weighted average to achieve the purpose of improving the accuracy.

Ghost Bottleneck module is an important part of a successful model because it allows sufficient or redundant information in the feature layer to always guarantee understanding of the input data. By choosing YOLOv5s as the modification object, the depth and width of the model can be compressed. In a sense, small model search for different data sets can be realized. Therefore, Ghost Bottleneck module is introduced into the Backbone module. Lightweight design does not remove redundancy, but with a lower cost of computing.

**Figure 5:**
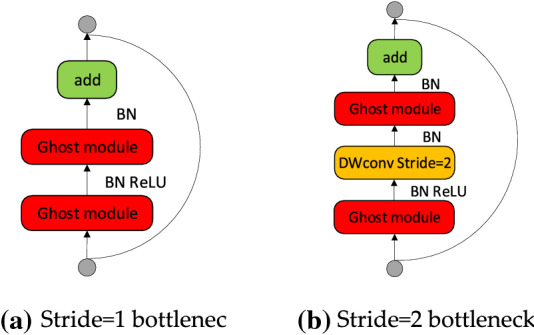
Ghost bottleneck.

The GhostBottleneck module is added to the Backneck module to reduce the overall size of the model without increasing network parameters, make the network better and faster understand redundant information and improve the accuracy of the model. The GhostBottleneck module is similar to the Basic Residual Block in ResNet. It has two structures^18^, namely, the bottleneck with Stride=1 and Stride=2, as shown in Fig. 5. Among them, the first Ghost module in Stride=1 is used as the extension layer, increasing the number of channels. The ratio of the number of output channels to the number of input channels is called the expansion ratio. The second Ghost module reduces the number of channels to match the shortcut path. Then, use shortcut to connect the input and output of the two Ghost modules. The second Ghost module in the GhostBottleneck module does not use ReLU, and the other layers apply batch normalization and ReLU activation functions after each layer. Corresponding to the case of Stride=2, it plays the role of down sampling in this model, realizes a fast path, and inserts a DWConv with Stride=2 between two ghost modules for connection.

### Improvement of neck network

In order to further compress network parameters and realize lightweight model design, the Neck layer in the original YOLOv5s is improved. It mainly replaces Conv in the original PANet module with DWConv. Different from the conventional convolution operation, depthwise convolution has a convolution kernel responsible for a channel, and a channel is convolved by only one Convolution kernel. The original BottleneckCSP module was replaced with Stride = 1 GhostBottleneck module, which saved a lot of calculation cost and achieved better model performance.

### Training platform and network initialization parameters

In order to facilitate detection, the network input size was set to 1751pix × 911pix, mainly because the particle board surface defect size is relatively small. The hardware and software parameters of PB-YOLOv5s in this study are shown in Table 3. According to the image feature information of particleboard surface defect dataset and GPU performance test, a better training effect is obtained when the Batch size is set to 16. Regularization is performed through the BN layer to update the weight of the model. Set the number of training epochs to 300.

**Table 3:** Hardware and software parameters of PB-Yolov5s.

Hardware environment		Software environment	
Memory	256 GB	System	Windows Server 2012 R2 Standard
CPU	Intel(R) Xeon(R) Gold6152 CPU@2.10 GHz	Environment configuration	Python 3.8.3
GPU	NIVIDIA Quadro RTX 8000		Pytorch dynamic development framework

### Various evaluation indexes

In this study, training set and test set are used to train and test the model. The loss functions used in the training of PB-YOLOv5s model mainly include classification loss (*L*_*classification*_), confidence loss (*L*_*confidence*_) and boundary box positioning loss (*L*_*CIoU*_)^19,20^. The calculation equations are as follows:

1 $$LOSS = L_{classification} + L_{confidence} + L_{CIoU}$$

2 $$L_{classification} = \sum\limits_{i = 0}^{{s^{2} }} {\ell_{i}^{obj} } \sum\limits_{j = 0}^{B} {\left[ {\left( {p_{i} \left( c \right) - \mathop {p_{i} }\limits^{ \wedge } \left( c \right)} \right)^{2} } \right]}$$

3 $$L_{confidence} = \sum\limits_{i = 0}^{{s^{2} }} {\sum\limits_{j = 0}^{B} {\ell_{i}^{obj} \left[ {\left( {C_{i} - \mathop {C_{i} }\limits^{ \wedge } } \right)^{2} } \right]} } + \lambda_{noobj} \sum\limits_{i = 0}^{{s^{2} }} {\sum\limits_{j = 0}^{B} {\ell_{i}^{noobj} \left[ {\left( {C_{i} - \mathop {C_{i} }\limits^{ \wedge } } \right)^{2} } \right]} }$$

In Eq. (2), $\ell_{i}^{obj}$ means to judge whether there is an *object* center in grid III. If the grid contains an *object* center, it is responsible for predicting the category probability of the *object*. In Eq. (3), $C_{i}$ represents the confidence score, $\mathop {C_{i} }\limits^{ \wedge }$ represents the intersection of the prediction boundary box and the basic facts, and $\lambda_{noobj}$ represents the weight of the *classification* error.

Based on the CIoU loss, this study solved the problems of aspect ratio, center distance and overlap area, as shown in Eqs. (4)–(6). Where, *v* represents the coincidence degree of the two frame aspect ratios, $\frac{{\omega^{gt} }}{{h^{gt} }}$ represents the aspect ratio of the *Ground truth* and the aspect ratio of prediction. Where, *v* represents the coincidence degree of the two frame aspect ratios, $\frac{{\omega^{gt} }}{{h^{gt} }}$ represents the aspect ratio of the ground truth, and $\frac{\omega }{h}$ represents the aspect ratio of prediction. Where α is a positive trade-off parameter, the overlapping area factor has higher priority in regression, especially in the case of nonoverlapping.

4 $$L_{CIoU} = 1 - IoU + \frac{{\rho^{2} \left( {b,b^{gt} } \right)}}{{c^{2} }} + \alpha \nu$$

5 $$IoU = \frac{{\left| {b \cap b^{gt} } \right|}}{{\left| {b \cup b^{gt} } \right|}}$$

6 $$\nu = \frac{4}{{\pi^{2} }}\left( {\arctan \frac{{\omega^{gt} }}{{h^{gt} }} - \arctan \frac{\omega }{h}} \right)^{2}$$

7 $$\alpha = \frac{\nu }{{\left( {1 - IoU} \right) + \nu }}$$

In this study, training indexes such as Precision, Recall, mAP and F1 score were used to evaluate the surface defect recognition performance of particleboard, as shown in the formula below. Precision is to evaluate whether the prediction of particleboard surface defects is accurate, reflecting the proportion of real positive samples in the positive examples determined by the classifier. Recall is the evaluation and prediction of whether all surface defects of particleboard have been found, which reflects the proportion of correctly determined positive cases in the total positive samples. F1 score is the harmonic average of precision and recall. MAP is the mean average accuracy, which is used as an indicator to measure the detection accuracy in object decision.

## Results

### Recognition results of surface defects of particleboard by PB-YOLOv5s model

**Figure 6:**
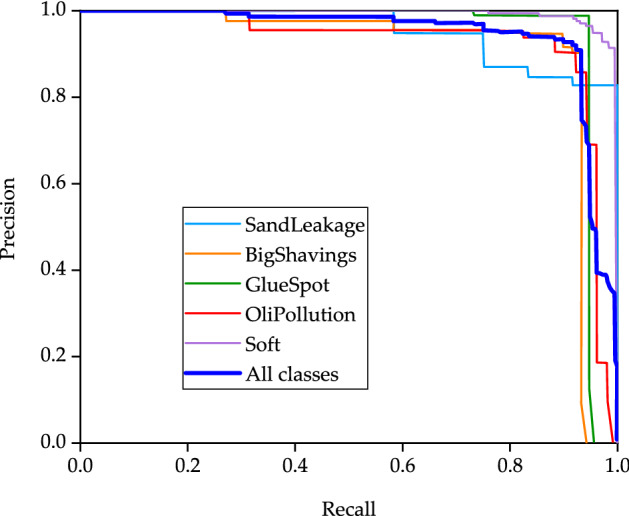
PR curve.

**Figure 7:**
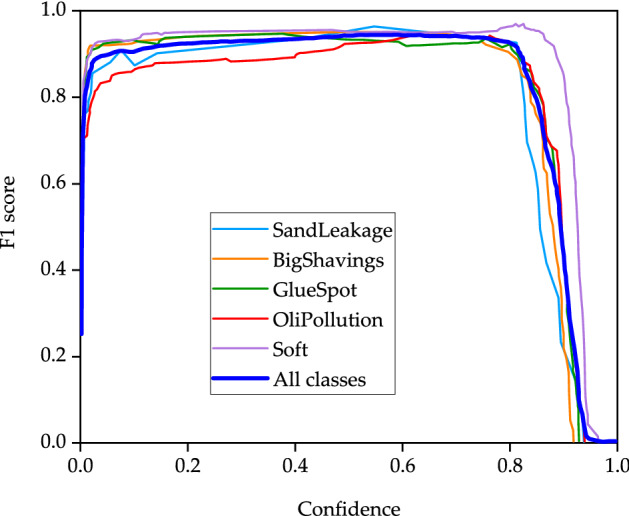
F1 score curve.

**Figure 8:**
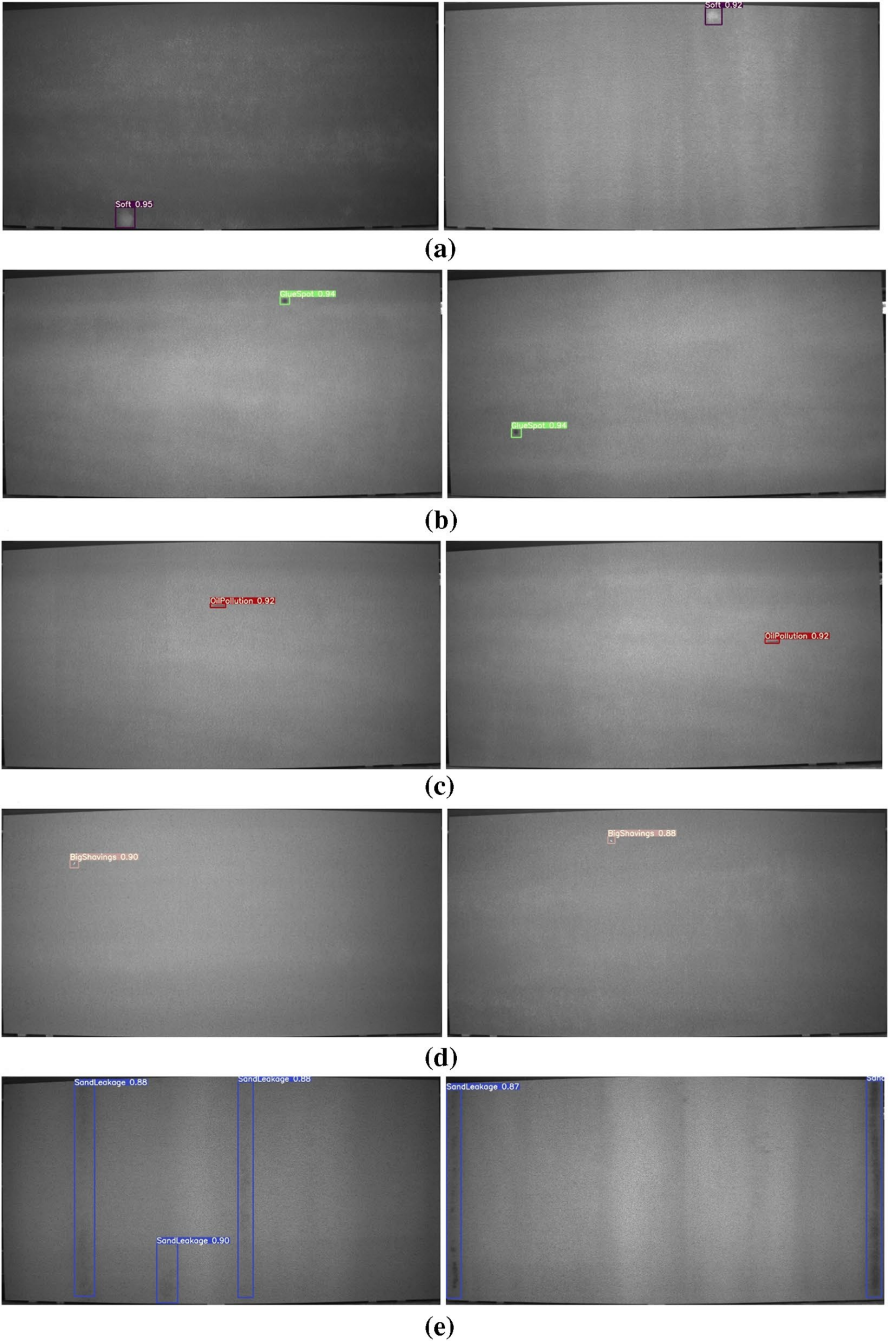
Surface defect detection results of particleboard based on PB-YOLOv5s algorithm: (**a**) Soft defect, (**b**) GlueSpot defect, (**c**) OilPollution defect, (**d**) BigShavings defect, (**e**) SandLeakage defect.

The purpose of YOLO target detection is the coexistence of accuracy and real-time. The most important thing is to improve the detection speed, that is, PB-YOLOv5s is the object of the following research and discussion. In order to verify the effectiveness of the proposed method, PB-YOLOv5s is used to train the images of particleboard surface defects (Bigshavings, SandLeakage, OliPollution, GlueSpot and Soft). The PR curve of the trained model is shown in Fig. 6, the F1 score image of the corresponding model is shown in Fig. 7, the recognition result image of the corresponding five types of defects is shown in Fig. 8, and the recognition results and indicators of the corresponding five types of defects are shown in Table 4.

**Table 4:** Identification results of particleboard surface defects.

Class	Precision (%)	Recall (%)	F1 score (%)	mAP@.5 (%)	mAP@.5:.95 (%)
SandLeakage	88.8	98.5	93.4	94.9	80.7
BigShavings	94.6	89.2	91.8	91.1	57.2
GlueSpot	90.7	94.6	92.6	94.4	62.1
OilPollution	89.2	92.2	90.7	92.9	57.4
Soft	92.8	97.9	95.3	99.0	81.7
All	91.2	94.5	92.8	94.4	67.8

The PR curve reflects the relationship between accuracy and recall. The purpose is to test the impact of different particle board surface defect types on the performance of the model. The closer the PR curve is to the upper right corner, the better. It can be seen that Soft defects are closer to the upper right corner, indicating that the precision and recall indexes of Soft defects are high. Because the color and size of Soft defects and particleboard are very different, it is easy to identify. BigShavings and GlueSpot defects are slightly worse than soft defects. The reason for this phenomenon is that the size of BigShavings and GlueSpot defects is too small, resulting in low precision and recall. The color of SandLeakage defect is almost the same as that of particleboard, which will have a certain interference on identification. In Fig. 6, the curve of SandLeakage defect begins to decline when the precision is 0.9 and the recall is 0.75. When the precision is 0.8, the recall value begins to remain unchanged.

From the detection results of F1 score curve, it can be seen that the F1 score values of Soft defect, BigShavings defect and GlueSpot defect image training model are higher than those of the model combined with five types of defect images. The F1 score of SandLeakage defect and OliPollution defect is slightly lower than that of the other three types of defects. The reason is that the edges of these two types of defects are not obvious, and they are in a gradual transition with the normal part, which is difficult to define in the transition area, so there are some differences in the detection results.

Table 4 shows the specific identification results of the proposed model for five types of particleboard surface defects, precision, recall, F1 score, mAP@.5, mAP@.5:.95 values were 91.22%, 94.5%, 92.8%, 94.4% and 67.8% respectively. Among them, the Soft defect recognition effect is the best, and the five indicators are 92.8%, 97.9%, 95.3%, 99.0% and 81.7% respectively, which is also directly related to the number of soft defect samples and defect shape. The overall detection accuracy of the model is high, and each index basically reaches more than 90%, which can meet the accuracy requirements of particle board surface defect detection.

There is a certain gap in the appearance of surface defects of different kinds of particleboard. Figure 8 shows the identification results of five types of defects. The purple box represents the Soft label, the green box represents the GlueSpot label, the red box represents the OilPollution label, the orange box represents the BigShavings label, the blue box represents the SandLeakage label, and the number next to the label represents the confidence of the predicted particleboard surface defects. In Fig. 8., it is obvious that the model can accurately identify defects, all defects are not missed, the detection results of five types of defects are high, and the confidence range is 0.87–0.95. The confidence scores of soft defects, GlueSpot defects and OilPollution defects are above 0.92, and Soft defects even reach 0.95. The confidence of SandLeakage defect and BigShavings defect is low, ranging from 0.87 to 0.9, which is related to the size and color of the defect itself. High confidence and robustness mean that particle board surface defects can be detected accurately and have relatively reliable performance.

### Comparison of PB-YOLOv5 and YOLOv5 evaluation indexes

In this study, eight models with different versions such as PB-YOLOv5s, YOLOv5s, PB-YOLOv5m, YOLOv5m, PB-YOLOv5l, YOLOv5l, PB-YOLOv5x and YOLOv5x are compared. Among them, s, m, l and x represent the increase of model depth in turn.

**Figure 9:**
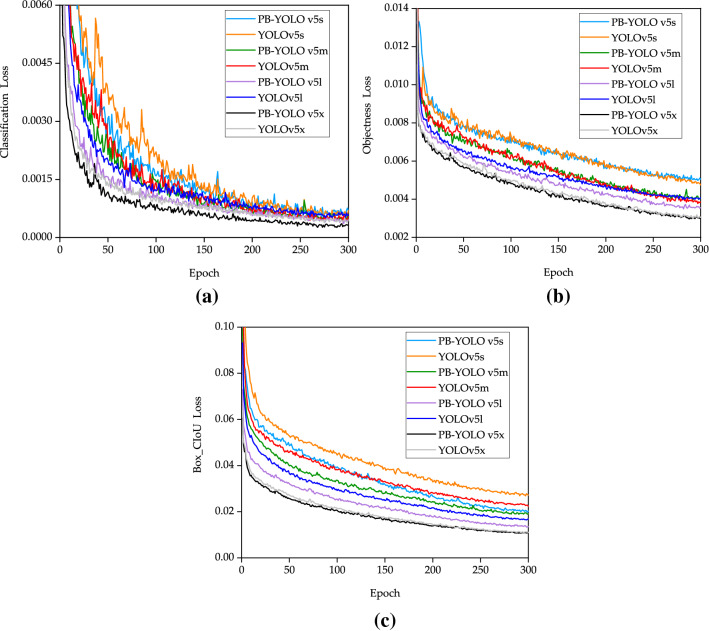
Comparison of loss functions under different models: (**a**) Classification loss curve, (**b**) Objectness loss (Confidence loss) curve, (**c**) Box_CIoU (Bounding box loss) curve.

Figure 9 shows the training process under different models, using classification loss function and objectness loss (confidence loss) function and box_compared with the CIoU loss (bounding box loss) function, the loss value represents the difference between the predicted value and the real value. The classification loss function is the average loss of classification, and the value is inversely proportional to the classification effect. The objectness loss function is the loss mean of the target detection confidence, and the value is inversely proportional to the target confidence. The Box_CIoU loss function is the mean value of the CIoU loss function, and the value is inversely proportional to the recognition effect of the prediction box. As shown in Fig. 9, the convergence loss of PB-YOLOv5 model in the training process is better than that of the improved YOLOv5 model. PB-YOLOv5 model can better locate the target position in the training process. At the begin of training, the value of the loss function of 50 epochs decreased rapidly. When the epoch reached 150, the loss value basically tended to be stable. PB-YOLOv5 model sets the loss function through the output and label information to update the network parameters. The convergence position of each loss function is less than 0.05, and the robustness of the model is good, so as to realize the effective prediction of the model.

**Figure 10:**
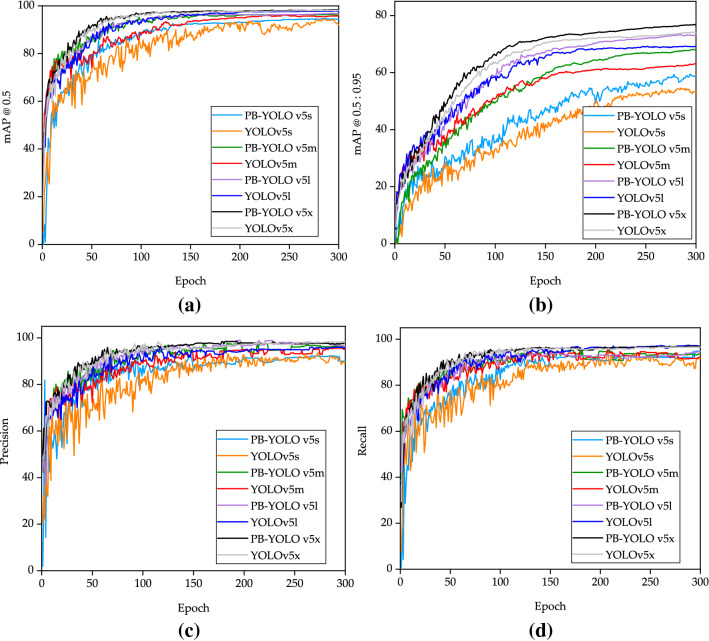
Comparison of evaluation indexes under different models: (**a**) mAP@.5 curve, (**b**) mAP @.5:.95 curve, (**c**) Precision curve, (**d**) Recall curve.

As shown in Fig. 10c,d, the precision curves and recall curves of several models are shown. It can be seen from the comparison curve that the curve of PB-YOLOv5 model rises gently without obvious oscillation. However, YOLOv5s in the rising stage of the curve, the amplitude fluctuation of the curve is obvious, and this phenomenon continues to the end. It shows that the detection effect is greatly improved by improving the model and adding attention mechanism. As shown in Fig. 10a,b and Table 5, the mAP value of PB-YOLOv5 model is higher than that of YOLOv5 model before improvement. The higher mAP value also indicates that the model performance after training is better. The mAP@0.5 values of PB-YOLOv5 model are all over 94%, and the mAP values of the improved model are 1.5% higher than that of the pre-improved model. As can be seen from the above figure, PB-YOLOv5 is well trained and no fitting phenomenon has ever occurred.

**Table 5:** Comparison table of model training weight and training time.

Model	Model weight size (MB)	Training time (hours)	Detection of single image time (s)	Model parameter size	mAP @ 0.5 (%)	mAP @ 0.5: 0.95 (%)
PB-YOLOv5s	5.4	7.801	0.031	2.55 × 10^6^	94.4	58.4
YOLOv5s	14.4	9.613	0.047	7.07 × 10^6^	92.1	53.2
PB-YOLOv5m	14.0	15.221	0.043	6.78 × 10^6^	96.3	67.8
YOLOv5m	42.5	20.077	0.055	2.11 × 10^7^	95.8	63.0
PB-YOLOv5l	28.4	25.028	0.051	1.39 × 10^7^	96.6	73.1
YOLOv5l	93.8	54.328	0.060	4.67 × 10^7^	97.8	69.3
PB-YOLOv5x	49.7	57.583	0.057	2.46 × 10^7^	98.3	76.7
YOLOv5x	167.0	144.246	0.069	8.73 × 10^7^	98.0	74.1

As shown in Table 5, it can be found that the weight of the PB-YOLOv5s model is only 5.4MB, only about 1/3 of that of YOLOv5s, and the model has the least parameters. The low inference time and small model size mean that the model is very fast to detect and can still be explored to a greater extent using the higher computing power of the GPU. The results show that the detection results of PB-YOLOv5 model are higher, and the weights and parameters of the model are smaller than those of YOLOv5 before improvement, and the detection speed of PB-YOLOv5 is also the fastest.

### Comparison between this study and other target detection algorithms

The indexes of PB-YOLOv5s model designed in this paper and the most commonly used target detection algorithm on particleboard surface defect dataset are compared, as shown in Table 6. It can be seen that the PB-YOLOv5s model has a high mAP value on the particleboard surface defect dataset, and the weight size is the smallest. The weight file size is only 1/44 of that of YOLOv4, and the detection speed of PB-YOLOv5s is the fastest. The results show that the PB-YOLOv5 model has a high accuracy, the detection speed can meet the real-time requirements, and achieves a good balance between the detection speed and accuracy, and is easy to deploy. It provides a technical reference for the application of the model in the terminal equipment in the future, and promotes the development of particle board surface defect detection equipment.

**Table 6:** Performance comparison of four object detection networks.

Model	Detection of single image time (s)	Model weight size (MB)	Model parameter size	mAP (%)
Our model (PB-YOLOv5s)	0.031	5.4	2.55 × 10^6^	94.40
YOLOv4	0.069	235.0	6.39 × 10^7^	89.71
YOLOv3	0.087	244.0	6.27 × 10^7^	86.42
Faster RCNN	0.256	512.0	2.40 × 10^6^	81.35

## Discussion

The difficulty of particle board surface defect detection lies in high real-time performance and large amount of calculation, which occupies a lot of detection time. Although many achievements have been made in the current field of wood surface defect detection algorithms, there is little research on the real-time detection of particleboard surface defects, and the traditional target detection algorithm is adopted. In order to verify the recognition performance of the algorithm presented in this paper, the recognition results of the algorithm presented in this paper are compared with the recognition results of Wang et al. and Guo et al. Among them, the research results of Wang et al. and Guo et al. were the best in the field of detection, and there was no detailed data on the execution time of the algorithm in the rest of the literature.

As shown in Table 7, Mean Average precision values of the four models are all high, which are 94.4%, 98.3%, 94.3% and 92.2%, respectively. The detection accuracy of traditional target detection algorithm is lower than that of Deep learning target detection algorithm. The Traditional target detection algorithm requires image preprocessing before image detection, barrel correction of distorted image obtained by camera, and then pixel traversal, threshold comparison, gray operation of the whole image, which takes a long time. For example, Wang et al.^21^ used THE TLD method to detect the surface defects of particleboard, which was mainly composed of three parts: detection module, tracking module and learning module. These three modules took a long time. Guo et al.^10^ adopted GLCMHC algorithm for detection. The calculation time of feature parameters was 0.052s, and the Clustering time was 0.511s. The total time of detecting a single defective image was 0.563s. Due to the long time, it is only suitable for laboratory research and cannot be put into production. GLCMHC algorithm detection is an early research achievement of our team members. The problem was that when the threshold of a certain area reaches the specific value of defect, that is, the board judged as defective will not traverse downward and directly output instructions. However, when there were no defects on the whole surface of particleboard, the algorithm traverses and calculates the pixels of the whole image, which takes nearly 3s, affecting the real-time detection.

**Table 7:** Comparison between this study and other particle board surface defect detection algorithms.

Model	Detection of single image time (s)	Mean average precision (mAP) (%)	Target detection category
Our model (PB-YOLOv5s)	0.031	94.4	Deep learning target detection
Our model (PB-YOLOv5x)	0.057	98.3	Deep learning target detection
Tracking-Learning Detection Technology (TLD)	0.100	94.3	Traditional target detection
Gray Level Co-occurrence Matrix and Hierarchical Clustering (GLCMHC)	0.563	92.2	Traditional target detection

Considering that the surface defects of particleboard have particularly high requirements for real-time performance, the current enterprises of particleboard production line clearly stipulate that the detection line speed is 1500mm/s, which requires the detection system to detect whether there are defects on the surface of particleboard within 3s and then fall into the corresponding area for classification and stacking. If the detection cannot be completed within 3s, the particleboard will cross the classification gate and fail to be classified and detected, and the phenomenon of missing detection will affect the product quality. Therefore, a lightweight particle board surface defect detection method is adopted in this study, which not only meets the necessary conditions of real-time, but also meets the requirements of accuracy. It can be seen from Table 7. that PB-YOLOv5 series has the best comprehensive performance, and the detection time is greatly improved compared with the two methods of traditional target detection, which fully meets the requirements of particle board surface defect detection. At this stage, due to the uneven quality of raw materials and inconsistent working conditions, the color and defect form of particleboard in each batch are different. This model can effectively identify defect types, overcome many factors of working conditions and sheet materials, and has strong generalization ability, without the need to readjust parameters. It can realize the automatic detection of board defects, reduce the phenomenon of missing and wrong detection of defective boards, reduce the economic loss of board defects to enterprises, and improve the automation level and operation efficiency of particleboard production line. In contrast, the traditional target detection algorithm needs to readjust the threshold parameters through the particleboard quality of each batch and the factory environment. Adjusting the threshold was a complex process, which greatly reduces the detection efficiency.

## Conclusions

Based on the real-time algorithm of particleboard surface defect detection based on deep learning, this research proposes and constructs the PB-YOLOv5s network model, trains the network model with a large amount of data, focuses on the rapid defect detection and identification algorithm, and meets the requirements of high real-time performance, low missed detection rate and low false detection rate of the online detection system. In order to reduce the overall size of the model, GhostBottleneck module, SELayer module and DWConv module are introduced to make the network better and faster understand redundant information, save model parameters and operation cost, improve the robustness of the model to spatial layout and object recognition, and obtain a lightweight model. The test results show that PB-YOLOv5s model is better than the original YOLOv5 model in various test indexes, and the weight file is only 1/3 of YOLOv5s, which is more convenient to be applied in mobile devices and embedded devices. In order to improve the application scope of the algorithm and particle board surface defect detection equipment, the defect sample database will be established while the system is applied for a long time in the future, and the sample database will be collected and improved in time. In the later training model, the improved data set is added to train a better and more complete defect detection model, so that the system can correctly detect all defects.

## References

[1] Chang L, Guo W, Lv B, Zhang Y (2017). Development status and trends of China’s Particleboard Industry. China Wood-Based Panels.

[2] Wei ZF, Xiao SH, Jiang GZ, Wu S, Cheng G (2021). Research on surface defect detection of wood-based panels based on deep learning. China Forest Prod. Ind..

[3] Way D, Kamke F, Sinha A (2018). Influence of specimen size during accelerated weathering of wood-based structural panels. Wood Mater. Sci. Eng..

[4] Zhou HD, Ding T, Lu B, Jiang XT, Liu HL (2015). Analysis of labor usage and up grade equipment on laminating flooring. Wood Process. Mach..

[5] Chacon, M. & Alons, G. Wood defects classification using A SOM/FFP approach with minimum dimension feature vector. In *Advances in Neural Networks—ISNN 2006*, *Third International Symposium on Neural Networks, Chengdu, China, May 28—June 1* 1105–1110. https://link.springer.com/chapter/10.1007%2F11760191_161 (2006).

[6] He T, Liu Y, Xu C, Zhou X (2019). A fully convolutional neural network for wood defect location and identification. IEEE Access..

[7] Xie Y, Wang J (2015). Study on the identification of the wood surface defects based on texture features. Optik.

[8] Zhang, Y. X., Zhao, Y. Q., Liu, Y., Jiang, L. Q., & Chen, Z. W. Identification of wood defects based on LBP features. In *Proceedings of the IEEE 2016 35th Chinese Control Conference (CCC), Chengdu, China. 27–29 July* 4202–4205 (2016).

[9] Li S, Li D, Yuan W (2019). Wood defect classification based on two-dimensional histogram constituted by LBP and local binary differential excitation pattern. IEEE Access.

[10] Guo H, Wang X, Liu CZ, Zhou YC (2018). Research on defect extraction of particleboard surface images based on gray level co-occurrence matrix and hierarchical clustering. Sci. Silvae Sin..

[11] Yang WX, Jin LW, Tao DC, Xie ZC, Feng ZY (2016). Dropsample: A new training method to enhance deep convolutional neural networks for large-scale unconstrained handwritten Chinese character recognition. Pattern Recognit..

[12] Shi JH (2020). Real-time leak detection using an infrared camera and faster R-CNN technique. Comput. Chem. Eng..

[13] Wang YT, Wang KF, Zhu ZX, Wang FY (2020). Adversarial attacks on faster R-CNN object detector. Neurocomputing.

[14] Huang H (2020). Single spectral imagery and faster R-CNN to identify hazardous and noxious substances spills. Environ. Pollut..

[15] Xu BB (2020). Automated cattle counting using mask R-CNN in quadcopter vision system. Comput. Electron. Agric..

[16] Lawal MO (2021). Tomato detection based on modified YOLOv3 framework. Sci. Rep..

[17] Li SW, Gu XY, Xu XR, Xu DW (2021). Detection of concealed cracks from fround penetrating radar images based on deep learning algorithm. Constr. Build. Mater..

[18] Han, K. *et al*. GhostNet: More features from cheap operations. In *Proceedings of the 2020 IEEE/CVF Conference on Computer Vision and Pattern Recognition (CVPR), Seattle, WA, USA, 13–19 June*. 1577–1586. 10.1109/CVPR42600.2020.00165 (2020).

[19] Redmon, J., Divvala, S., Girshick, R. & Farhadi, A. You only look once: Unified, real-time object detection. In *Proceedings of the 2016 IEEE Conference on Computer Vision and Pattern Recognition (CVPR), Las Vegas, NV, USA, 27–30 June* 779–788. 10.1109/CVPR.2016.91 (2016).

[20] Redmon, J. & Farhadi, A. YOLO 9000: Better, faster, stronger. In *Proceedings of the 2017 IEEE Conference on Computer Vision and Pattern Recognition (CVPR), 21–26 July* 6517–6525. 10.1109/CVPR.2017.690 (2017).

[21] Wang CC, Liu YQ, University NF (2018). Detecting surface defects of particleboard based on tracking-learning detection technology. China Wood Ind..

